# Tumor Microenvironment: Extracellular Matrix Alterations Influence Tumor Progression

**DOI:** 10.3389/fonc.2020.00397

**Published:** 2020-04-15

**Authors:** Sylvie Brassart-Pasco, Stéphane Brézillon, Bertrand Brassart, Laurent Ramont, Jean-Baptiste Oudart, Jean Claude Monboisse

**Affiliations:** ^1^Université de Reims Champagne Ardenne, SFR CAP-Santé (FED 4231), Laboratoire de Biochimie Médicale et Biologie Moléculaire, Reims, France; ^2^CNRS UMR 7369, Matrice Extracellulaire et Dynamique Cellulaire - MEDyC, Reims, France; ^3^CHU Reims, Service Biochimie-Pharmacologie-Toxicologie, Reims, France

**Keywords:** cancer, microenvironment, extracellular matrix, matrikines, integrins, proteases

## Abstract

The tumor microenvironment (TME) is composed of various cell types embedded in an altered extracellular matrix (ECM). ECM not only serves as a support for tumor cell but also regulates cell–cell or cell–matrix cross-talks. Alterations in ECM may be induced by hypoxia and acidosis, by oxygen free radicals generated by infiltrating inflammatory cells or by tumor- or stromal cell-secreted proteases. A poorer diagnosis for patients is often associated with ECM alterations. Tumor ECM proteome, also named cancer matrisome, is strongly altered, and different ECM protein signatures may be defined to serve as prognostic biomarkers. Collagen network reorganization facilitates tumor cell invasion. Proteoglycan expression and location are modified in the TME and affect cell invasion and metastatic dissemination. ECM macromolecule degradation by proteases may induce the release of angiogenic growth factors but also the release of proteoglycan-derived or ECM protein fragments, named matrikines or matricryptins. This review will focus on current knowledge and new insights in ECM alterations, degradation, and reticulation through cross-linking enzymes and on the role of ECM fragments in the control of cancer progression and their potential use as biomarkers in cancer diagnosis and prognosis.

## Introduction

The tumor microenvironment (TME) is a complex structure composed of a large variety of cell types embedded in a modified extracellular matrix (ECM), with bidirectional communication between cells and ECM macromolecules to determine tumor progression and metastatic dissemination. The communication may involve cell–cell contacts but may also be controlled by intact ECM macromolecules or by several of their domains released by limited proteolysis and called matrikines or matricryptins. In this review, we will focus on ECM alterations occurring in TME, on the role of released matrikines in the control of cancer progression, and on the potential use of ECM fragments as biomarkers for cancer diagnosis and prognosis.

## Tumor Microenvironment: An Active Player in Cancer Progression

Tumors are diverse by the nature of their TME composition, stromal cell proportion, and activation states. TME undergoes transformations during tumor progression as a result of tissue remodeling. TME comprises a wide variety of cell types such as fibroblasts, endothelial cells, pericytes, and immune and inflammatory cells. These different cells elicit cross-talks leading to cell activation and differentiation and alterations in ECM structural and biological properties facilitating tumor cell proliferation, invasion, and metastatic dissemination. Within the TME, different T cell and B cell populations infiltrate invasive tumors and draining lymphoid organs (1). Tumor-associated macrophages (TAMs) are either tissue-resident or derived from bone marrow or spleen and play an important role in tumorigenesis regulation by facilitating cell migration, invasion, and metastasis (2). Tumor cells lead to the recruitment of neutrophils in tumorigenesis sites by secreting chemokines and interleukin (IL)-8. Infiltration by neutrophils appears to confer a poor prognosis (3). A dominant cellular component is fibroblasts that exert a key role in cancer progression and metastasis. Fibroblasts are usually quiescent and become activated to differentiate into myofibroblasts, also called cancer-associated fibroblasts (CAFs) (4). The main progenitors of CAFs come from resident fibroblasts, but CAFs can also come from smooth muscle cells, pericytes, or from bone marrow-derived mesenchymal cells leading to a heterogeneous cell population (5–7). Growth factors, secreted by tumor cells and infiltrating immune cells, largely govern stromal fibroblast recruitment. Transforming growth factor (TGF)β, platelet-derived growth factor (PDGF), and fibroblast growth factor (FGF)2 are key mediators of fibroblast activation. CAFs become synthetic machines that produce TME components creating an ECM structure as well as metabolic and immune reprogramming of TME. CAF secretome includes growth factors [epidermal growth factor (EGF), bone morphogenetic protein (BMP), FGF, or TGFβ] and some chemokines such as C-X-C motif ligand (CXCL)12 or stroma-derived factor (SDF)-1, which recruit circulating endothelial progenitor cells (4). These soluble factors, in conjunction with the angiogenic switch and several miRNAs, stimulate endothelial cells and their associated pericytes to develop tumor angiogenesis or lymphangiogenesis (2).

## Metabolic Alterations in the Tumor Microenvironment

During the local growth of tumor, the surrounding vessels fail to meet the high demand of oxygen leading to hypoxic areas within the tumor and TME (8). Prolyl-hydroxylases are responsible for the labeling of hypoxia-inducible factors (HIFs) to be degraded by 26S proteasome. Under hypoxic conditions, prolyl-hydroxylases are inhibited, leading to the stabilization of HIFs that induces the expression of various genes implicated in tumor progression. Moreover, hypoxic responses include the unfolded protein response (UPR) and mammalian target of rapamycin (mTOR) signaling (9). mTOR signaling, through the phosphoinositide 3-kinase (PI3K)/Akt pathway, largely contributes to the regulation of cell survival, growth, and metabolism through phosphorylation of the eukaryotic initiation factor 4E-binding protein 1 (4E-BP1 protein) and ribosomal protein S6 kinase (10). HIF-1 is also a key regulator of the metabolic switch. By inducing specific gene expression, it alters the cellular metabolism, increasing glycolysis and lactate production (11, 12). Lactate arises from glycolysis which takes place under hypoxic conditions, but in tumors, glycolysis can also take place in oxygenated areas (8).

Nicotinamide adenine dinucleotide phosphate (NADPH) oxidase of inflammatory cells generates oxidative stress. Superoxide ions are converted into hypochlorous acid (HOCl) by myeloperoxidase and into OH^∙^ radicals. Tumor cells with a high metabolism also release reactive oxygen species (ROS) and promoted ROS production in CAFs. ROS induce oxidative stress in TME and activate HIF-1 and nuclear factor (NF)-κB pathways, leading to an increase in autophagy (7). ROS also induce strong alterations in DNA, cell membrane, and ECM components. For example, collagen I is partially degraded by ROS and becomes more susceptible to proteolytic cleavage (13). Among proteases, neutrophils or TAMs secrete matrix metalloproteinase (MMP)-8 and-9 as well as neutrophil elastase that collaborates with CAF-secreted proteases to degrade ECM.

**Figure 1:**
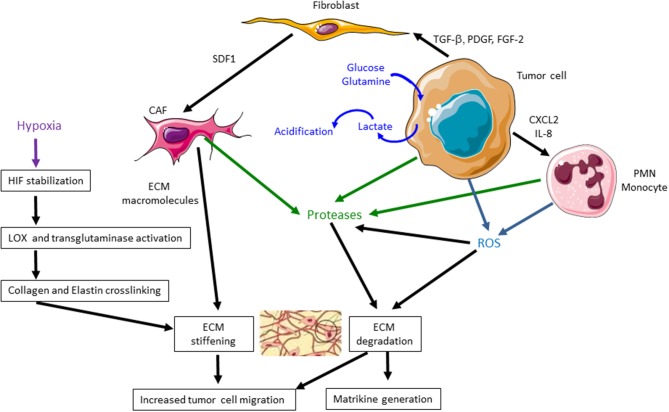
Main metabolic and extracellular matrix (ECM) alterations in the tumor microenvironment (TME) during tumor progression. During cancer progression, tumor cells increase lactate production, leading to an acidification of TME. Tumor cells, cancer-associated fibroblasts (CAFs), polymorphonuclear leukocytes (PMNs), and monocytes secrete proteases, such as matrix metalloproteinases (MMPs), that degrade ECM and release matrikines. CAFs induce a higher secretion of ECM macromolecules that leads to an excessive deposition of ECM components. Tumor cells, PMNs, and monocytes produce reactive oxygen species (ROS) that degrade ECM components and particularly collagen I, facilitating tumor cell migration. They also stimulate the production of MMPs. Hypoxia also induces hypoxia-inducible factor (HIF) stabilization, lysyl oxidase (LOX) and transglutaminase activation, collagen and elastin cross-linking leading to ECM stiffening. These events favor tumor cell migration and cancer progression.

Main metabolic alterations of TME are summarized in Figure 1.

## Extracellular Matrix Alterations in the Tumor Microenvironment

Another important feature of TME is the composition and organization of ECM, whose mechanical properties affect cell behavior. The ECM is mainly secreted by CAFs which produce more ECM proteins than normal fibroblasts. It is composed of various macromolecules including collagens, glycoproteins (fibronectin and laminins), proteoglycans, and polysaccharides with different physical and biological properties. Interstitial matrix, primarily synthesized by stromal cells, is rich in fibrillar collagens and proteoglycans. CAF secretome analyses show an increased secretion of bone morphogenetic protein (BMP)1, thrombospondin-1, and elastin interface 2 (7, 14). Several splice variants of fibronectin ED-A and ED-B and tenascins C and W may be secreted by CAFs (15). Interstitial ECM is highly charged and hydrated and greatly participates in the tensile strength of tissues. Stiffness of neoplastic tumors is strongly higher than adjacent normal tissues. Cancer cells, CAFs, and TAMs, stimulated by hypoxia, modulate together ECM within the TME through an excessive deposition of structural components such as collagens, as well as cross-linking enzymes of the lysyl oxidase (LOX) and transglutaminase families, particularly LOX-1, LOXL-2, and transglutaminase-2 (16, 17). Collagen and elastin fibers are reoriented and cross-linked by LOX and transglutaminase, resulting in larger and more rigid fibrils that facilitate cell migration (18, 19). Figure 1 summarizes the main ECM alterations in TME.

## Extracellular Matrix Breakdown by Migrating Cancer Cells

A decisive hallmark in cancer progression is the crossing of ECM and basement membrane (BM) by cancer cells. To penetrate the ECM, cancer cells secrete a number of proteolytic enzymes of the MMP family. BMs are specialized ECMs which are more compact and less porous. They present a distinct composition with collagen IV and laminin interconnected networks and proteoglycans such as perlecan. Several other types of collagen are associated to the BM, collagens XV, XVIII, and XIX. During ECM-barrier crossing, proteases release soluble and active fragments referenced in Table 1, called matrikines or matricryptins which may control cancer progression.

**Table 1:** ECM fragments affect the main hallmarks of cancer progression.

**ECM bioactive fragments**	**Parent molecule**	**Generating enzymes**	**Receptors**	**Biological activity**
**Collagen fragments**				
Type IIB procollagen NH2 propeptide	Type IIB collagen	ADAMTS-3 (20)	αvβ3, αvβ5 integrins (21)	↗ EC and tumor cell death (chondrosarcoma, cervical and breast cancer) (21) through programmed cell necrosis (22)
Arresten (α1 chain NC1 domain)	Type IV collagen	Cathepsin S (23) MT1-MMP, MT2-MMP (24)	α1β1 integrin (25, 26)	↘ Angiogenesis and tumor growth (melanoma, glioblastoma, colorectal and lung cancer, squamous cell carcinomas) (25) ↘ FAK/c-Raf/MEK-1/2/ERK-1/2/p38 MAPK pathways in EC↗ EC apoptosis through bcl-xl*/*bax ratio modulation (25)
Canstatin (α2 chain NC1 domain)	Type IV collagen	Cathepsin S (23) MT1-MMP, MT2-MMP (24)	α1β1, αvβ3, αvβ5 integrins (27)	↘ Angiogenesis and tumor growth (ocular, lung, breast, oral squamous cell, esophageal carcinoma, gastric, ovarian, pancreatic, prostate, and colorectal cancer (28) ↘ VEGF-A/VEGFR-1-2 signaling pathway in squamous cell carcinoma (29) ↗ Apoptosis in cancer cell and EC through bcl-2bcl-xl*/*bax ratio modulation (30) ↘ Caspase 8 and 9 activation in EC (27)
Tumstatin (α3 chain NC1 domain)	Type IV collagen	MMP-9 (31)	αvβ3, αvβ5 integrins (32)	↘ Angiogenesis and tumor growth (melanoma, glioma, osteosarcoma, breast, colon, prostate and lung cancer, gastric, hepatocellular, and squamous cell carcinoma (33, 34)
54–132 amino-acid sequence				54–132 amino-acid sequence:↗ G1 arrest, ↗ caspase-3 activation and ↘ FAK/PI3K/Akt/mTOR pathway in ECs (35)
185–203 amino-acid sequence				185–203 amino-acid sequence :↘ melanoma and EC migration through a decrease in MMP-2, uPA, t-PA (36)
Tetrastatin (α4 chain NC1 domain)	Type IV collagen		αvβ3 integrin (37)	↘ Tumor growth (melanoma, glioma, osteosarcoma, breast, colon, prostate and lung cancer, gastric, hepatocellular and squamous cell carcinoma (37–40) ↘ FAK/PI3K/Akt pathway and ↘ MMP-2 in tumor cells (37, 38)
Lamstatin (α5 chain NC1 domain)	Type IV collagen			↘ Angiogenesis (41) and lung cancer growth (42, 43)Unknown molecular mechanism
Hexastatin (α6 chain NC1 domain)	Type IV collagen			↘ Angiogenesis and tumor growth (Lewis lung carcinoma and spontaneous pancreatic insulinoma) (44)Unknown molecular mechanism
Vastatin (NC1 domain of collagen VIII alpha 1 chain)	Type VIII collagen			↘ EC proliferation and tumor growth and metastasis in murine hepatocellular carcinoma models (45) ↘ PcK1, JAG2, and c-Fos, ↘ Notch/AP-1 pathway (46)
Restin (NC10 domain of collagen XV)	Type XV collagen			↘ EC migration, renal carcinoma growth (47) and breast cancer metastasis (48) ↘ ATF3 activity by direct interaction (49) ↘ EMT through p-73 binding, mir-200a/b increase and ZEB1/2 inhibition in breast cancer cells (48)
Endostatin (20-kDa C-terminal fragment of collagen XVIII)	Type XVIII collagen		α5β1 integrin; caveolin-1 (50)	↘ Angiogenesis, lymphangiogenesis and tumor growth (51) ↗ Src-kinase pathway, ↘ RhoA GTPase activity; ↘ Ras/c-Raf/p38/Erk-1 pathway in EC (52, 53)
				Frizzled domain (FZC18): ↘ Wnt/β-catenin pathway (54)
NC1 XIX	Type XIX collagen	Plasmin (55)	αvβ3 integrin (56)	↘ Melanoma cell migration, invasion, tumor growth and angiogenesis (56, 57) ↘ MMP-14 (57) in melanoma↘ FAK/PI3K/Akt/mTOR pathway in melanoma cells (57)
**Elastin fragments**				
VG-6 (VGVAPG)	Elastin	Proteinase 3, cathepsin G (58), MMP-7,9,12 (59), neprilysin (60)	ERC, αvβ3 and αvβ5 integrins, galactin-3 (61), RPSA (62)	↗ Angiogenesis (63) and tumor growth in melanoma models (62, 64, 65) ↗ MT1-MMP, ↗ PI3K/Akt/NO synthase, ↗ NO/cGMP/Erk1/2 pathways in EC (66) ↗ IL-1β through NF-κB pathway in melanoma cell (67) ↗ MMP and plasminogen activation cascades in cancer cells
AG-9 (AGVPGLGVG)	Elastin	Proteinase 3, cathepsin G (58), MMP-7,9,12 (59), neprilysin (60)	RPSA (62)	↗ Tumor growth in a melanoma model (62) ↗ Tumor cell migration, invasion through MMP and plasminogen activation cascades
**Laminin fragments**				
IKVAV (α1 chain fragment)	Laminin-111		α3β1 and α6β1 integrins (68)	↗ Angiogenesis, tumor growth, and metastasis (68) ↗ bone marrow mesenchymal stem cell proliferation by activating MAPK/ERK1/2 and PI3K/Akt signaling pathways (69) ↗ t-PA in melanoma cells (68)
AG73 (RKRLQVQLSIRT from α1 chain)	Laminin-111		Syndecans 1, 2, and 4 (68)	↗ Angiogenesis and tumor growth (68) ↗ Rac1 and ERK1/2 signaling pathways (70)
YIGSR (β1 chain fragment)	Laminin-111		67 KD receptor (68)	↘ Tumor growth and metastasis (68) Unknown mechanism
C16 (KAFDITYVRLKF from γ1 chain)	Laminin-111		αvβ3 and α5β1 integrins (68)	↗ Tumor growth (68) ↗ MMP-9 production in melanoma cells (68)
γ2 chain N-terminal fragment	Laminin 332	MMP-2, cathepsin S, MT1-MMP (71)	α3β1 integrin, CD-44 (71)	↗ Angiogenesis, tumor growth and metastasis (71) Unknown mechanism
α3 chain C-terminal fragment	Laminin 332	Plasmin, MMP-2, MT1-MMP, C-proteinase, mTLD, BMP-1 (71)	α3β1 and α6β1 integrins (71)	↗ Angiogenesis, tumor growth (71) Unknown mechanism
A5G27 (RLVSYNGIIFFLK from α5 chain)	Laminin 511		Cell surface glycans (72)	↘ Breast tumor cell proliferation↗ 4T1.2 experimental pulmonary metastasis (72) Unknown mechanism
**Fibronectin fragments**				
Anastellin (type III module)	Fibronectin			↘ Angiogenesis, tumor growth and metastasis (73) ↗ p38 MAPK activation in EC (74)
**Proteoglycans fragments**				
Metastatin	Aggrecan	ADAMTS (75)		↘ Growth, migration, angiogenesis of melanoma and prostate cancer (76) Unknown mechanism
EndorepellinLG3 fragment (C-terminal fragment of Endorepellin)	Perlecan	MMP-7 (77)Cathepsin L and BMP-1-Tolloid-like proteases (78)	α2β1 integrin (79)	↘ EC proliferation and migration, angiogenesis, tumor growth (78–84) ↘ VEGF-A/VEGFR pathway in EC (79) ↗ autophagy through Peg3 activation in EC (79, 85)
Versikine	Versican	ADAMTS (86)	TLR2 (34)	↗ Immunogenicity in myeloma (87, 88) ↗ IL-1β, IL-6 expression by myeloma-associated macrophages through both Ppl2 kinase-dependent or -independent pathways (88)
Lumcorin (SSLVELDLSYNKLKNIP)L9M (ELDLSYNKLK) Lumikine/LumC13 (YEALRVANEVTLN)	Lumican		α2β1 integrin (89), MMP-14 (90, 91), ALK5/TGFβR1 (92)	↘ Growth, migration, angiogenesis in melanoma and breast cancer (93–96) ↘ FAK/Akt/ERK pathway↘ MMP-14 proteolytic activity (90, 97) ↗ keratocytes migration (92, 98)
**Synstatins**				
SSTN 92-119, SSTN 82-130, SSTN 210-240	Syndecan-1		αvβ3, αvβ5 and α3β1 integrins, HER2, VEGFR2 (co-receptors of ectodomain) (34, 99–103)	↘ Angiogenesis in breast cancer (104–106)Depend on HER2- and EGFR-coupled mechanism (104)
SSTN87-131	Syndecan-4		EGFR, α3β1 integrin (co-receptors of ectodomain) (34)	↘ Cell motility (104)Depend on HER2- and EGFR-coupled mechanism (104)
**Glypican fragments**				
Glypican-3 derived peptide	Glypican-3		Wnt	↗ Cell proliferation, migration and invasion in hepatocellular carcinoma (107) ↗ Wnt/β-catenin, Hedgehog, and YAP pathway (108–110) ↗ Macrophage recruitments in tumor (108) ↗ EMT (108)
**Has**				
HA oligosaccharides	HA		CD44 (111)	Alters tumor growth, metastatic potential, and progression in prostate, colon, breast, and endometrial cancers (112, 113, 165)LMW HA promotes angiogenesis (114)HMW HA decreases angiogenesis, induces EMT (114)

*4E-BP1 protein, eukaryotic initiation factor 4E-binding protein 1; ADAMTS, a disintegrin and metalloproteinase with thrombospondin motifs; AP-1, activator protein 1; ATF, activating transcription factor; ALK5, TGFβ type I receptor kinase; BMP, bone morphogenetic protein; cGMP, cyclic guanosine monophosphate; EC, endothelial cell; ECM, extracellular matrix; EGFR, epidermal growth factor receptor; EMT, epithelial–mesenchymal transition; ERC, elastin receptor complex; ERK, extracellular signal-regulated kinase; FAK, focal adhesion kinase; HA, hyaluronan; HER2, human epidermal growth factor receptor-2; HMW-HA, high-molecular-weight HA; IL, interleukin; JAG2, jagged canonical Notch ligand 2; LMW-HA, low-molecular-weight HA; MAPK, mitogen-activated protein kinase; MEK, MAPK/ERK kinase; MMP, matrix metalloproteinase; mTLD, mammalian Tolloid; mTOR, mammalian target of rapamycin; NF, nuclear factor; NO, nitric oxide; PI3K, phosphoinositide 3-kinase; RPSA, ribosomal protein SA; TLR, Toll-like receptor; t-PA, tissue-type plasminogen activator; uPA, urokinase-type plasminogen activator; VEGF, vascular endothelial growth factor; VEGFR, vascular endothelial growth factor tyrosine kinase receptor; TGFβ, transforming growth factor β*.

## Extracellular Matrix-Derived Fragments Influence Tumor Progression

**Figure 2:**
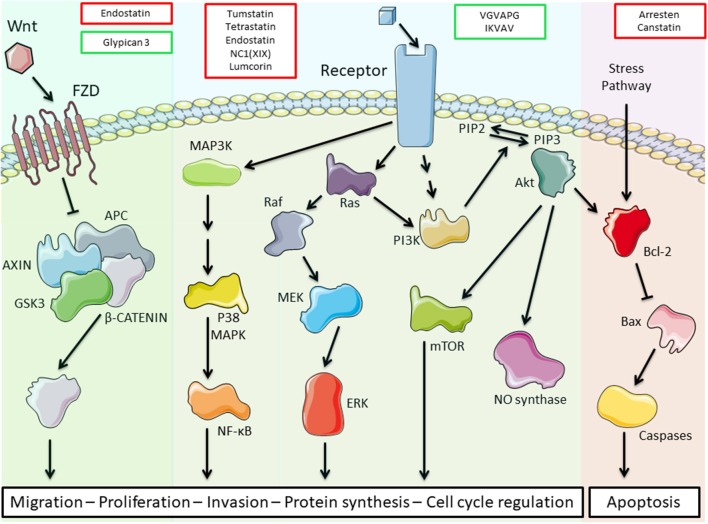
Schematic representation of the main transduction pathways altered by extracellular matrix (ECM) bioactive fragments. Bioactive fragments stimulating the pathway are outlined in green, and fragments with inhibitory activity are outlined in red. Endostatin inhibits the Wnt/β-catenin pathway, while glypican-3 triggers this pathway. Tumstatin, tetrastatin, endostatin, NC1(XIX), and lumcorin inhibit the phosphoinositide 3-kinase (PI3K)/Akt/mammalian target of rapamycin (mTOR) pathway through integrin binding while VGVAPG and IKVAV activate this pathway through elastin receptor complex (ERC) and integrin binding, respectively. VGVAPG and IKVAV also activate the mitogen-activated protein kinase (MAPK) pathways. Arresten and canstatin activate the Bcl-2 pro-apoptotic pathway through integrin binding.

The different matrikines derived from ECM macromolecules, collagens, glycoproteins, or proteoglycans may exert either pro- or anti-tumorigenic properties in various cancer models (Table 1). We and others demonstrated that collagen IV-derived matrikines (canstatin, tumstatin, and tetrastatin) and collagen XIX-derived matrikine act through binding to α3β1, α5β1, or αVβ3 integrins. The binding elicits an inhibition of the focal adhesion kinase (FAK)/PI3K/Akt/mTORC1 pathway, which is one of the main intracellular pathways involved in TME metabolic alterations. The inhibition leads to a decrease in the proliferative and invasive properties of tumor cells in various cancer models (27, 33, 38, 56). The main receptors, biological activities, and molecular mechanisms identified for ECM bioactive fragments are reported in Table 1 and are illustrated in Figure 2.

## Extracellular Matrix Fragments as Tumor Biomarkers

During cancer progression, an excessive ECM remodeling by proteinases, especially MMPs, is observed, and small ECM fragments are released into the circulation. The levels of these fragments may represent a measure of tumor activity and invasiveness and could be proposed as biomarkers (115). Serum and biofluid biomarkers are easy to collect, noninvasive, low cost, and can be followed over the course of the disease. Identification of new biofluid biomarkers may help in early detection, diagnosis, disease monitoring, and in individual treatment selection and thus on patient outcome. However, the low concentrations of ECM-derived fragments in body fluids remain a limitation to the development of these biomarkers in daily practice.

### Collagens

Type I collagen is a major ECM component susceptible to proteinase degradation during cancer progression. Type I collagen cross-linked carboxyterminal telopeptide (ICTP) measurement in patient sera appears to be useful for bone metastasis screening in lung cancer patients, including stage III–IV non-small-cell lung cancer (NSCLC) or extensive disease (ED) small-cell lung cancer (SCLC) (116). ICTP level in serum from patients with esophageal squamous cell carcinoma significantly correlates with tumor progression variables, including TNM stages (≥T2, N1, and M1), TNM stage ≥II, and maximal tumor length greater than 50 mm (117). A high level of ICTP in preoperated patient serum appears to be an important marker of better prognosis in triple-negative breast cancer and luminal-B-like [human epidermal growth factor receptor (HER)2-negative] subtypes (118). The elevation of the cross-linked N-telopeptide of type I collagen (NTx) appears positively related with the development and progression of bone metastasis in lung cancer (119). NTx serum concentration may also have a prognostic value in patients with prostate cancer at diagnosis (120). A high level of serum NTx (>22 nmol BCE/L) is correlated with a reduction in overall survival (OS) in patients with NSCLC (121).

In the follow-up of patients with radical resection of colorectal carcinoma, the N-terminal peptide of type III procollagen (marker of ECM synthesis) was reported as an early prognostic indicator of recurrence (122).

The serum level of tumstatin is significantly higher in patients with NSCLC compared to healthy patients (123).

The levels of markers reflecting type I (C1M), type III (C3M), and type IV (C4M, C4M12) collagen degradation by MMPs were significantly elevated in serum of ovarian or breast cancer patients compared to healthy controls (124).

Type VI collagen expression is correlated with various pro-tumorigenic events. Levels of type VI collagen α1 and α3 chain fragments, derived from MMP proteolysis, appear higher in serum from cancer patients (breast, colon, gastric, ovarian, pancreas, prostate cancer, NSCLC, SCLC, melanoma) compared to healthy patients and have promising diagnostic accuracy (125). Type VI collagen α3 chain circulating fragment levels were significantly higher in the serum of pancreatic ductal adenocarcinoma patients compared to healthy patients or patients with benign lesions (126).

Elevated serum endostatin levels were found in various human cancers including colorectal cancer (127), soft tissue sarcoma (128), and advanced-stage nasopharyngeal carcinoma (129). They are correlated with a favorable outcome in acute myeloid leukemia (130). On the contrary, high serum endostatin levels are associated with enhanced ECM degradation and poor patient outcome in patients with bladder cancer (131) and with non-Hodgkin lymphoma (132). Determination of soluble vascular endothelial growth factor tyrosine kinase receptor (sVEGFR)-1 and endostatin levels may be useful in the diagnosis of malignant pleural effusions in patients with lung cancer (133). Preoperative serum VEGF and endostatin levels may be used for evaluating the biological behavior, invasion, and metastasis of gastric, hepatocellular, and colorectal carcinoma (134).

### Elastin

Elastin fragments, released by proteases, are increased in the serum of stage I–IV NSCLC patients compared to healthy controls. These results suggest the use of elastin fragments as potential biomarkers (135), but further validations in clinical trials are needed.

### Laminins

Laminins were reported to promote tumor progression. The serum level of LNγ2 fragments increases according to the T classification of head and neck squamous cell carcinoma (HNSCC) and decreases after the use of curative treatments. The level of LNγ2 fragments in serum may be useful to predict response to treatment of patients with HNSCC (136). The presence of soluble laminin fragments (ULN) corresponding to the N-terminal domain of the β2 chain was measured in urine of healthy subjects and patients with tumor. Mean level of ULN in lung tumor patients is significantly higher than that in healthy subjects (137). Serum laminin P1 fragment was studied in patients with SCLC and NSCLC and in normal subjects. The serum concentration of laminin P1 was elevated in 58.9% of SCLC and in 11.5% of NSCLC patients compared to healthy subjects. Median value in SCLC patients was significantly higher than that in NSCLC patients and in normal subjects (138). Urine laminin P1 measurement allows to discriminate between invasive and noninvasive urothelial cell carcinoma of the bladder (139).

### Proteoglycans

The cleavage of proteoglycans like aggrecan and versican by a disintegrin and metalloproteinase with thrombospondin motifs (ADAMTS) in epithelial ovarian cancer has been demonstrated and is considered of prognostic value (75).

Perlecan fragments in the serum of prostate cancer patients were correlated with overall MMP-7 staining levels in prostate cancer tissues. Domain IV fragments of perlecan were highlighted in stage IV patient sera, but not detected in normal patient sera, suggesting that perlecan is degraded during metastasis. The association of perlecan fragments in sera and MMP-7 expression in tissues reflects prostate cancer invasivity (77). In breast cancer, the level of the endorepellin LG3 fragment in serum was significantly lower in breast cancer patients compared to healthy subjects. This suggests the endorepellin LG3 fragment as a new potential serological biomarker in breast cancer (140).

NSCLC patients presenting tumors with a low concentration of sulfated glycosaminoglycans (GAG) and high proteoglycan (PGs) levels presented better overall survival compared to patients with a high concentration of sulfated GAG and low expression of proteoglycans. These data suggest that matrix PGs could be considered as biomarkers in lung cancer (141).

Versican has been shown to be a potential biomarker in different cancers such as hepatocellular carcinoma (142), colon cancer (143), and recently in ovarian cancer (144). Hope et al. (145) provide a rational for testing versican proteolysis as a predictive and/or prognostic immune biomarker.

Lumcorin, a lumican-derived peptide mimics the inhibitory effect of lumican in melanoma progression (97). Lumikine, another lumican-derived peptide, promotes the healing of corneal epithelium debridement (92). These peptides might be putative cancer biomarkers but, to our knowledge, there are up to now no data in the literature describing lumican-derived peptides as biological markers in cancer.

Syndecan-1 was reported to play an immunomodulatory function in the polarization of CD4^+^ T helper (Th) cells that were isolated from the TME of inflammatory breast cancer (IBC) and non-IBC patients (99). These results suggest that syndecan-1 expression in tumor could offer therapeutic potential in breast cancer. Remarkably, syndecan-1 seems to be overexpressed in inflammatory breast cancer, making it a potential biomarker.

New biomarkers such as syndecan-2 gene methylation (with improved detection sensitivity and specificity at lower costs) should lead to a great improvement in colorectal cancer screening. Syndecan-2 gene methylation was reported as a frequent event in precancerous lesions and appears detectable in bowel lavage fluid to identify patients with colorectal cancer (146, 147).

Syndecan-3- and aggrecan-peptides were recently described as novel biomarkers for the detection of epithelial ovarian cancer (144).

Syndecan-1 and syndecan-4 are described as independent indicators in breast carcinomas (148). Peptides based on interaction motifs in syndecan-1 and syndecan-4, named synstatins or SSTN peptides, are potential therapeutic agents for carcinomas depending on the HER2 and epidermal growth factor receptor (EGFR) pathway for their invasion and survival (104).

Glypican-1 detected in exosomes was suggested as a putative biomarker for early detection of pancreatic (149–154) and colorectal cancer (155, 156).

Glypican-3 is an important player in the Wnt, Hedgehog, and YAP signaling cascades involved in cancer cell proliferation and migration (108, 109). It is overexpressed in hepatocarcinoma and lung carcinoma and was reported as a poor prognosis marker in hepatocarcinoma. Glypican-3 represents a promising immunotherapeutic target. Different GPC3-targeting therapies have been developed: the use of humanized anti-GPC3 cytotoxic antibodies, the treatment with peptide/DNA vaccines, immunotoxin therapies, and genetic therapies (107, 157–162).

The involvement of CD44 and hyaluronan (HA) and the interaction of both molecules were demonstrated in numerous cancers (Table 1) and suggest their potential as biomarkers. HA molecules may exert distinct effects depending on their size and concentration. High-molecular-weight HAs (HMW HAs) are involved in cell proliferation and tissue development, whereas low-molecular-weight HAs (LMW HAs) enhance angiogenesis. Serum level of LMW HA in patients with breast cancer was correlated with lymph node metastasis, and LMW HA was suggested as a cancer biomarker (114). An increase in HA levels induces tumor growth in mice and is associated with poor prognosis in pancreatic ductal adenocarcinoma (PDAC) patients. The inhibition of HA synthesis/signaling or the depletion of HA in tumor stroma may be a promising therapeutic approach to fight against PDAC progression (112). HA was also reported to facilitate cell proliferation and invasiveness in malignant pleural mesothelioma (163) and in melanoma (164) and may be used as a biomarker for early diagnosis and management of these diseases (163–165).

## Conclusion

ECM fragments evidenced peripheral tissue proteolysis by cancer cells and could control cancer progression by exerting both anti-angiogenic and anti-tumorigenic properties. We showed that ECM-derived bioactive fragments are able to inhibit major transduction pathways involved in TME alterations, such as the FAK/PI3K/Akt/mTORC1 pathway (Figure 2). They represent potent antitumor agents that might be useful in combination with conventional chemo-, immune-, and targeted therapies as part of personalized medicine. Moreover, they diffuse into the body and are easy to measure in the blood or body fluids and thus can represent valuable markers for the diagnosis and prognosis of numerous cancers.

## Author Contributions

SB-P, SB, BB, and JM contributed to manuscript writing. LR and J-BO contributed to manuscript revision. J-BO designed Figure 2. All authors approved the final version of the manuscript.

### Conflict of Interest

The authors declare that the research was conducted in the absence of any commercial or financial relationships that could be construed as a potential conflict of interest. The handling Editor declared a past co-authorship with one of the authors BB.

## Abbreviations

ADAMTS: a disintegrin and metalloproteinase with thrombospondin motifs
BMP: bone morphogenetic protein
CAF: cancer-associated fibroblast
ECM: extracellular matrix
ERC: elastin receptor complex
FGF: fibroblast growth factor
4E-BP1 protein: eukaryotic initiation factor 4E-binding protein 1
HER2: human epidermal growth factor receptor-2
LN: laminin
LOX: lysyl oxidase
MMP: matrix metalloproteinase
mTOR: mammalian target of rapamycin
PDGF: platelet-derived growth factor
ROS: reactive oxygen species
SDF 1: stroma-derived factor 1
SSTN: synstatin
sVEGFR1: soluble VEGF tyrosine kinase receptor 1
TAM: tumor-associated macrophage
TGFβ: transforming growth factor β
TME: tumor microenvironment.
